# Isolation and Characterization of Maize *PMP3* Genes Involved in Salt Stress Tolerance

**DOI:** 10.1371/journal.pone.0031101

**Published:** 2012-02-13

**Authors:** Jing Fu, Deng-Feng Zhang, Ying-Hui Liu, Sheng Ying, Yun-Su Shi, Yan-Chun Song, Yu Li, Tian-Yu Wang

**Affiliations:** 1 College of Biological Sciences, China Agricultural University, Beijing, China; 2 National Key Facility for Crop Gene Resources and Genetic Improvement (NFCRI), Institute of Crop Science, Chinese Academy of Agricultural Sciences, Beijing, China; Max Planck Institute for Chemical Ecology, Germany

## Abstract

Plasma membrane protein 3 (PMP3), a class of small hydrophobic polypeptides with high sequence similarity, is responsible for salt, drought, cold, and abscisic acid. These small hydrophobic ploypeptides play important roles in maintenance of ion homeostasis. In this study, eight *ZmPMP3* genes were cloned from maize and responsive to salt, drought, cold and abscisic acid. The eight ZmPMP3s were membrane proteins and their sequences in trans-membrane regions were highly conserved. Phylogenetic analysis showed that they were categorized into three groups. All members of group II were responsive to ABA. Functional complementation showed that with the exception of ZmPMP3-6, all were capable of maintaining membrane potential, which in turn allows for regulation of intracellular ion homeostasis. This process was independent of the presence of Ca^2+^. Lastly, over-expression of *ZmPMP3-1* enhanced growth of transgenic *Arabidopsis* under salt condition. Through expression analysis of deduced downstream genes in transgenic plants, expression levels of three ion transporter genes and four important antioxidant genes in ROS scavenging system were increased significantly in transgenic plants during salt stress. This tolerance was likely achieved through diminishing oxidative stress due to the possibility of *ZmPMP3-1's* involvement in regulation of ion homeostasis, and suggests that the modulation of these conserved small hydrophobic polypeptides could be an effective way to improve salt tolerance in plants.

## Introduction

Plant growth and development are affected by various abiotic stresses, such as high salinity, drought, low temperature and heavy metals. In particular, salinity is a global environmental challenge, affecting crop production over 800 million hectares, or a quarter to one third of all agricultural land on the earth [1]. Recently, physiological and genetic mechanisms of salt tolerance have been intensively investigated, and it is believed that high concentration of salts often causes ion imbalance and hyperosmotic stress to plants [2], [3].

Ion homeostasis is fundamental to physiological processes of living cells. The living cells often maintain high concentration of K^+^ and low concentration of Na^+^ in the cytosol, which is important for activities of many cytosolic enzymes. However, under salt stress, Na^+^ accumulates extremely in cells and consequently disrupts ion homeostasis. Thus, the maintenance of Na^+^ and K^+^ homeostasis is crucial under salt stress for plants to survive.

There are two kinds of mechanisms for plants to maintain ion homeostasis under salt stress. Firstly, at the organismal level, the ability of regulating Na^+^ uptake and transporting Na^+^ from roots to the shoots is critical in all plants. In saline soil, plant roots are inclined to minimize Na^+^ accumulation in plants. For instance, sodium influx of halophyte roots is much lower than non-halophyte roots because the width of the Casparion band is two or three times larger in halophytes than in non-halophytes, which effectively prevent the excessive Na^+^ from entering into the apoplastic space [4]. When the Na^+^ ion enters the apoplastic space of roots, the excessive Na^+^ is restricted to old tissues to prevent Na^+^ from accumulating in reproductive and delicate organs, which can lead to irreversible damage.

Secondly, at the cellular level, the maintenance of appropriate Na^+^ accumulation in cells is due to diffusion and active transport. Recently, some factors responsible for ion transport, such as nonselective cation channels (NSCCs), ion transporters and membrane-potential modulators, have been characterized. In plants, the NSCCs, which catalyze ion influx, can be divided into three groups according to their physical stimuli. These are respectively cyclic-nucleotide-gated NSCCs (CNGSs), amino-acid-gated NSCCs (AAG-NSCCs) and reactive-oxygen-species-activated NSCCs (ROS-NSCCs) [5]. Among these NSCCs, CNGSs are perhaps the best studied. Known examples include AtCNGS3, which localizes in root epidermal and cortical cells and contributes to Na^+^ uptake at the initial stage of salt stress [6], [7]. Other examples include AAS-NSCCs and ROS-NSCCs, which support the role of Ca^2+^ transporter [8]. Previous studies also revealed that there are several ion transporters that play important roles in retrieving intracellular ion homeostasis under saline conditions. As a plasma membrane Na^+^/H^+^-antiporter, SOS1 is an important tolerance determinant involved in the exclusion of sodium ions from cells [9], [10]. The transcription level of *SOS1* is up-regulated by salt stress but not by drought and cold stress [11]. In the presence of calcium, SOS3 activates the substrate phosphorylation activity of SOS2 [12], and then the SOS3/SOS2 complex in turn activates SOS1 probably *via* phosphorylation, which catalyzes sodium efflux from plant cells. Additionally, several other transporters are also involved in sodium ion transport, such as AtNHX1 and AtNHX2 [13].

Plasma membrane protein 3 (PMP3), a class of small molecular weight hydrophobic proteins in higher plants, responds to various stresses, such as low temperature, salt and dehydration. [14]–[16]. All these proteins are highly conserved at both sequential and structural levels and contain the common conservative domain of UPF0057. The PMP3 protein (Pmp3p) was also identified in yeast (*Saccharomyces cerevisiae*), which functions in the maintenance of plasma membrane potential. Deletion of *PMP3* increased Na^+^ and K^+^ sensitivity of yeast cells and resulted in excessive concentration of Na^+^ and K^+^ [16]. Complementation analysis in yeast revealed that plant homologues, such as *RCI2* in *Arabidopsis thaliana*, *Lti6a/b* in *Oryza sativa*, *AcPMP3-1* in sheep grass (*Aneurolepidium chinense*, a monocotyledonous halophyte), *PutPMP3-1* and *PutPMP3-2* in alkali grass (*Puccinellia tenuiflora*), are capable of restoring salt sensitivity of yeast mutant lacking *PMP3/SNA1* [16]–[22]. It was also demonstrated that AcPMP3 regulated cellular Na^+^ and K^+^ accumulation in the *Δnha1Δpmr2Δpmp3* yeast mutant under salt stress [22]. In *Arabidopsis*, eight *PMP3* homologues (*AtRCI2A-H*) have been identified [23]. Over-expression of *AtRCI2A* in *Arabidopsis* restricted Na^+^ uptake into cells and resultantly enhanced salt resistance. On the contrary, the disruption of *AtRCI2A* led to over accumulation of Na^+^ and increased Na^+^ sensitivity of *Arabidopsis* mutants [24], [25]. These reports indicate that *PMP3* homologues participate in maintaining intracellular ion homeostasis. However, due to the small size of these polypeptides, they are likely not ion transporters [22]. While it is generally agreed that Pmp3p are involved in cation uptake indirectly, the precise mechanism is currently unknown.

In maize (*Zea mays* L.), important genes that participate in regulation of ion homeostasis, such as *ZmCIPK16*, *ZmCBL4* and *ZmNHXs*, have been identified [26]–[28]. *ZmNHX* genes are likely to play a role in ion homeostasis regulation, as they are induced by salt stress in roots or stems [28]. Additionally, increased activities of Na^+^/H^+^-antiporter and H-pump enhance salt tolerance of maize seedlings [29]. Although it is known that *PMP3* genes likely play an important role in ion homeostasis regulation, there was no systemic collection and characterization of *PMP3* genes in maize. In this study, we systematically investigated functions of eight *ZmPMP3* genes. The result showed that these genes could complement the function of the yeast Pmp3p deletion mutant under salt stress. Over-expression of *ZmPMP3-1* enhanced salt tolerance of transgenic *Arabidopsis* plants. The up-regulations of ion transporters genes in transgenic plants, such as *NHX1*, *AVP1* and *AHA2*, are probably suggestive of the regulation of ion homeostasis by *ZmPMP3-1*.

## Results

### Isolation and sequence analysis of *ZmPMP3* family genes

**Figure 1:**
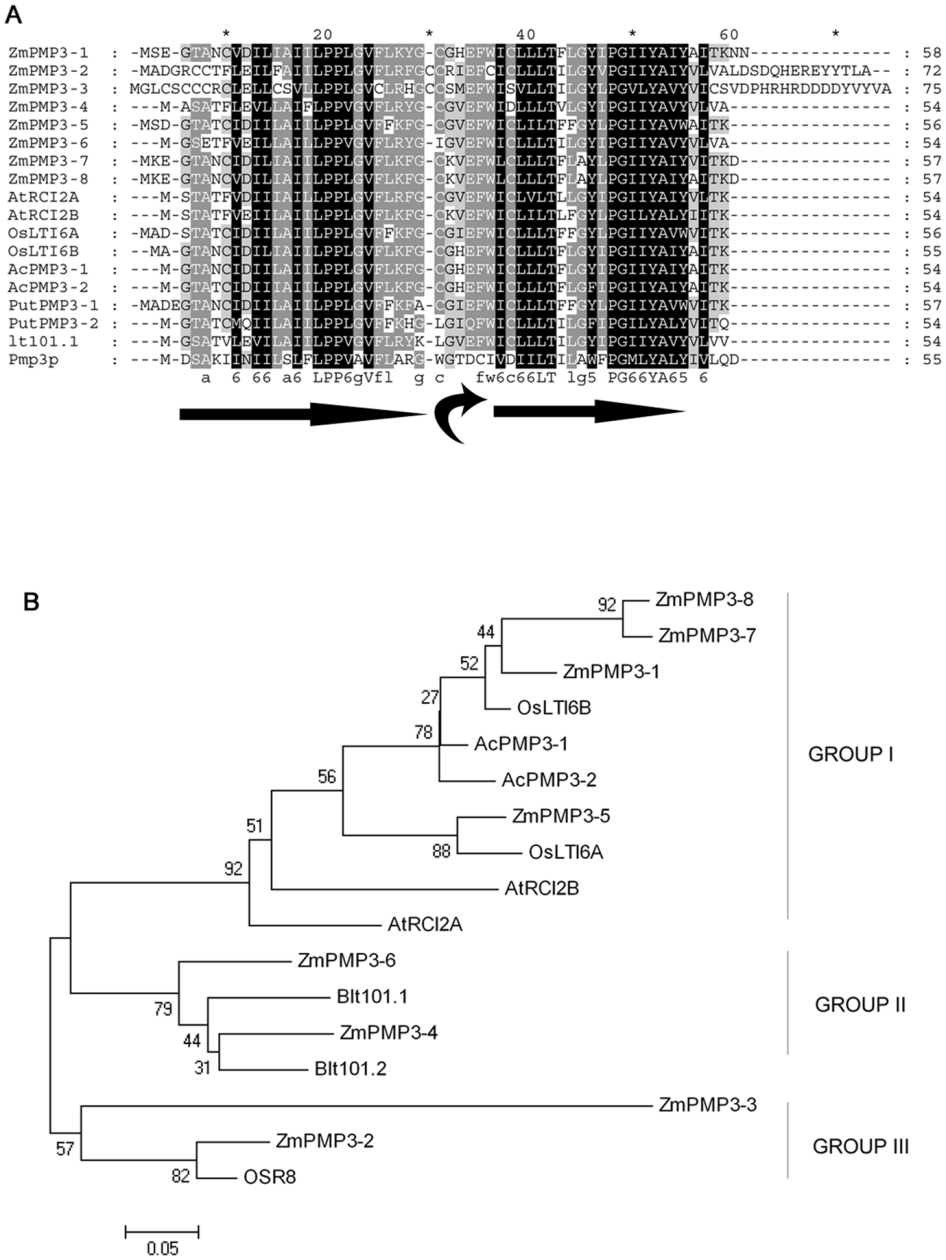
Comparison of ZmPMP3s and plasma membrane protein 3 related proteins. **A**) Alignment of deduced amino acid sequences of ZmPMP3s with *Arabidopsis* AtRCI2A/B (NP_187239.1, NP_187240.1), rice Oslit6a/b (AY607689.1, A2Y075.2), *Aneurolepidium chinense* AcPMP3-1/2 (BAD34658.1, BAD34659.1), *Puccinellia tenuiflora* PutPMP3-1/2 (BAG54793.1, BAG54794.1), *Hordeum vulgare* Blt101 (CAA80984.1). The amino acid identity in dark grey is larger than 75%, and the amino acid identity in light grey is larger than 50%. Straight arrows marked predicted transmembrane domains. The curved arrow indicated a putative loop structure. **B**) Phylogenetic analysis based on putative amino acid sequences of PMP3-related proteins in rice, *Arabidopsis*, *Hordeum vulgare*, *Aneurolepidium chinense*, and *Puccinellia tenuiflora*. The analysis was performed by using the MEGA 4.0 program with neighbor joining method and with 1000 replicates. Numbers on the figure 1B were bootstrap values.

To identify salt- or drought-stresses responsive genes, a cDNA library was constructed from salt-, drought-, and alkali-treated maize inbred line YQ7-96. An up-regulated EST (EC858820.1) homologous to *PMP3* was selected. After rapid amplification of cDNA 3′ end (3′RACE), a full-length sequence of *ZmPMP3-1* (EU364580) encoding a putative hydrophobic protein of 58 amino acids was obtained. There are seven other members of this gene family in maize according to the submitted sequences in GenBank, namely from *ZmPMP3-2* to *ZmPMP3-8* (Table 1). Open reading frames (ORFs) of the eight genes ranged from 165 bp to 228 bp and encoded peptides of 54 to 75 amino acids. Comparison among genomic and coding sequences revealed that the *ZmPMP3* genes, except *ZmPMP3-1*, harbored two exons separated by an intron of variable size. The ZmPMP3s encoded by these genes exhibited identities with each other from 42.17% to 95.38%. Particularly, the UPF0057 domain (LIVF-x-STAC-LIVF (3)-P-PF-LIVA-GAV-IV-x (4)-GKN) was conserved among these putative proteins of ZmPMP3 genes (Figure 1A). The results of the TMHMM 2.0 analysis showed that these putative proteins contained two transmembrane domains composed of alpha-helices, being linked by a putative turn structure. In addition, the orientations of N- and C- termini of the members were different (Figure S1) [30]. Comparison of the amino acid sequences of these eight genes revealed that the membrane spanning regions were highly conserved and the difference in protein lengths was caused by variable lengths of the C-terminal (Figure 1A).

**Table 1:** Identification of the *ZmPMP3* genes in maize.

Name^a^	Accession NO.^b^	Length^c^	BAC^d^	Bin^e^	Mr^f^	pI^g^
*ZmPMP3-1*	EU364580	58	AC206954	10.03	6390.71	5.54
*ZmPMP3-2*	EU959002.1	72	AC203412	2.06	8165.88	4.59
*ZmPMP3-3*	EU962407.1	75	AC186437	9	8385.11	5.37
*ZmPMP3-4*	EU954642.1	54	AC186651	1.03	5920.24	3.96
*ZmPMP3-5*	EU975274.1	56	AC203667	7.05	6172.54	4.36
*ZmPMP3-6*	EU976341.1	54	AC214362	6.01	5942.33	4.15
*ZmPMP3-7*	EU955642.1	57	AC208042.3	8.01	6391.91	6.25
*ZmPMP3-8*	EU971491.1	57	AC191649.3	3.04	6349.82	6.25

a Name of the identified *ZmPMP3* genes.

b Accession number of the *ZmPMP3* genes in GenBank.

c The number of the putative amino acids of the ZmPMP3 proteins.

d The BAC number of the *ZmPMP3* genes localized in B73 genome.

e Bin localizations of chromosomes of the *ZmPMP3* genes in B73 genome.

f Predicted molecular weights of the putative ZmPMP3 proteins.

g Predicted isoelectric points of the putative ZmPMP3 proteins.

Homologues of the ZmPMP3s were also present in many other plants species, such as *Arabidopsis*, *Oryza sativa* L., *Aneurolepidium chinense*, and *Puccinellia tenuiflora*. The maize ZmPMP3s showed sequential similarity with rice Oslti6b (AY607690) from 36% to 87.93%.

Cluster analysis revealed that ZmPMP3s could be divided into three groups (Figure 1B). The group I was composed of four members ZmPMP3-1, ZmPMP3-5, ZmPMP3-7 and ZmPMP3-8, which contained 56–57 amino acids and more closely related with AtRCI2A than other ZmPMP3s. In group II, ZmPMP3-4 and ZmPMP3-6 containing 54 amino acids. ZmPMP3-2 and ZmPMP3-3 belonged to group III.

With the completion of the B73 maize genome sequencing, the *ZmPMP3* genes were located on chromosomes by blasting in MaizeGDB database (http://www.maizegdb.org/). The eight genes were located on eight different chromosomes but chromosome 4 and 5 did not contain any *ZmPMP3* gene (Table 1).

### Subcellular localization of ZmPMP3s

**Figure 2:**
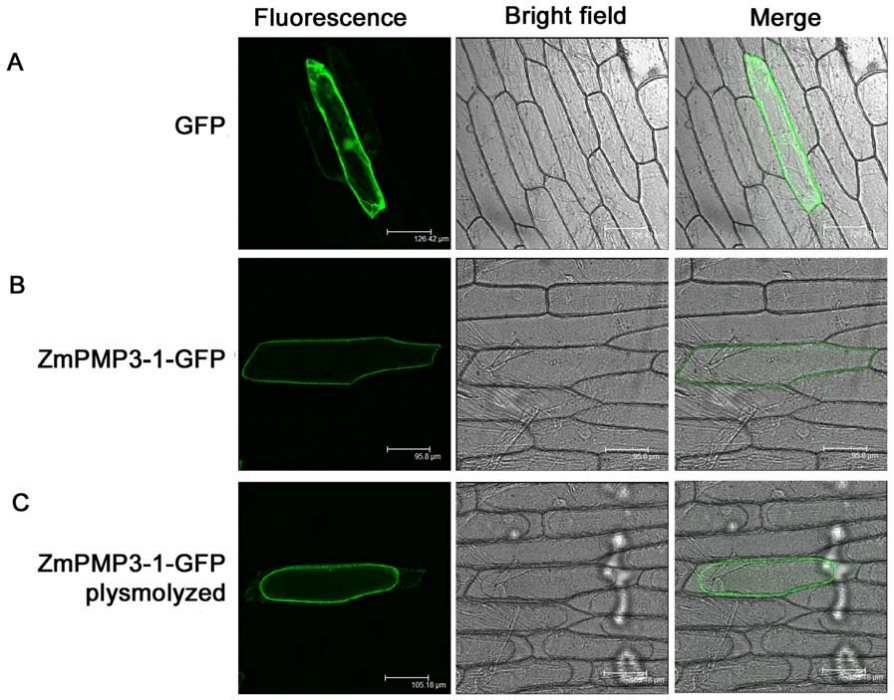
Subcellular localization of ZmPMP3-1-GFP fusion proteins in onion epidermal cells. **A**) Fluorescent microscopic images of GFP protein. **B**) Fluorescent microscopic images of nonplasmolyzed cells transiently expressing ZmPMP3-1-GFP fusion protein. **C**) Fluorescent microscopic images of plasmolyzed cells in 30% sucrose solution.

To investigate the localization of ZmPMP3s, the full-length coding regions without terminating codons of the *ZmPMP3* genes were cloned into the pBI221-GFP plasmid [31]. The ZmPMP3s-GFP fusion proteins were transiently expressed in onion epidermal cells and the subcellular localizations of these fusion proteins were visualized by confocal microscopy. As showed in Figure 2, GFP protein was found in the nucleus, cytoplasm, and cell membrane (Figure 2A, S2C). All of eight ZmPMP3-GFP fusion proteins showed GFP signals in the peripheral layer of cells, a location of which may correspond to either cell wall or plasma membrane. To further examine the location of ZmPMP3s, the epidermal cells expressing ZmPMP3-GFP fusion proteins were plasmolyzed by 30% sucrose solution. In these cells, the fluorescence was still detected in regions corresponding to the distorted plasma membrane (Figure 2B, C, S2A, and S2B), suggesting that the eight proteins were membrane proteins.

### Responses to various abiotic stresses

**Figure 3:**
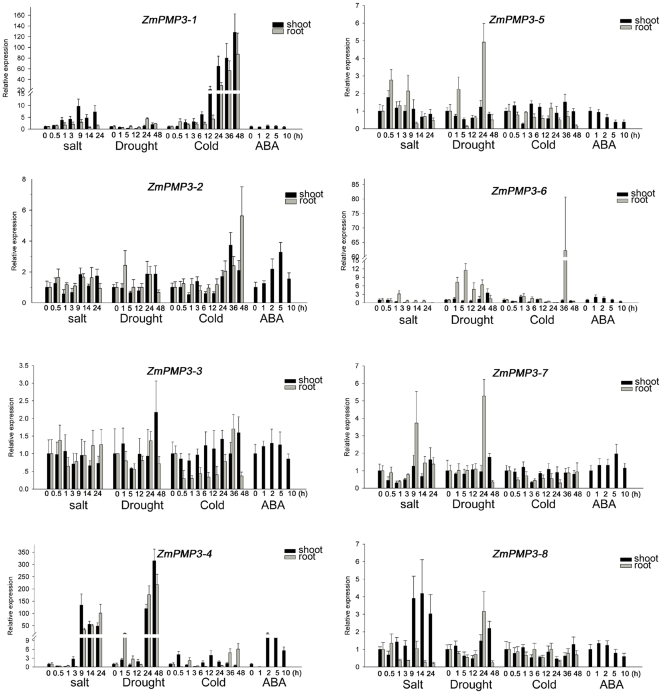
Expression patterns of the eight *ZmPMP3* genes in CN165 under various stresses. One-week old shoots and roots of maize seedlings, treated by 200 mM NaCl, 4°C, 30% PEG-6000, and 100 µM ABA, were harvested respectively. The qRT-PCR was performed to determine the relative expression levels of the eight genes under various treatment. The maize *GAPDH* gene was selected as internal control. Data from qRT-PCR experiments were analyzed according to the 2^−ΔΔCT^ method. Data represents the average of three independent experiments ±SE. when the relative expression levels go up to 2 fold or down to 0.5 fold, the difference of the expression is regarded as significant (P value<0.05).

The eight *ZmPMP3* genes displayed different expression patterns under abiotic stresses (Figure 3). Under salt treatment, *ZmPMP3-1* and *ZmPMP3-5* were up-regulated in shoot and root; *ZmPMP3-6* was only up-regulated in root and *ZmPMP3-8* was only up-regulated in shoot; *ZmPMP3-4* and *ZmPMP3-7* transcripts decreased slightly at 0.5 h or 1 h and then increased significantly at 9 h.

Under drought treatment, the transcript levels of *ZmPMP3-1*, *ZmPMP3-2*, *ZmPMP3-6*, *ZmPMP3-7* and *ZmPMP3-8* increased. The *ZmPMP3-7* transcript in root accumulated to maximal at 24 h followed by a reduction at 48 h. The transcript levels of *ZmPMP3-4* showed a minor peak at 1 h, and the accumulation increased significantly at 24 h. The *ZmPMP3-5* in root was repressed at 5 h followed by an increase with its peak at 24 h.

There were only four genes responsive to cold stress. The *ZmPMP3-1* and *ZmPMP3-2* transcripts in shoot and root were accumulated gradually under cold stress, while *ZmPMP3-4* and *ZmPMP3-6* were induced rapidly at 0.5 h or 1 h and then fell.

RT-PCR revealed that *ZmPMP3-1*, *ZmPMP3-5* and *ZmPMP3-6* were early responsive to these stresses. Under salt and drought conditions, the stronger inductions of *ZmPMP3-5* and *ZmPMP3-6* were observed only in root. While the expression of *ZmPMP3-3* was unaffected by those stresses.

*ZmPMP3-2*, *ZmPMP3-4*, *ZmPMP3-5* and *ZmPMP3-6* were responsive to exogenous ABA treatment but with different patterns. *ZmPMP3-2* and *ZmPMP3-6* were up-regulated under ABA treatment. The transcript levels of *ZmPMP3-2* and *ZmPMP3-6* increased to the maximum level at 5 h and 1 h, respectively, under ABA treatment. *ZmPMP3-5* was down-regulated. The transcript level of *ZmPMP3-4 was* dependent on time-point under ABA treatment since it decreased after 1 h and began to increase to the maximum level after 2 h. These results suggested that the four genes were regulated by ABA signal.

### Expression patterns in different tissues

**Figure 4:**
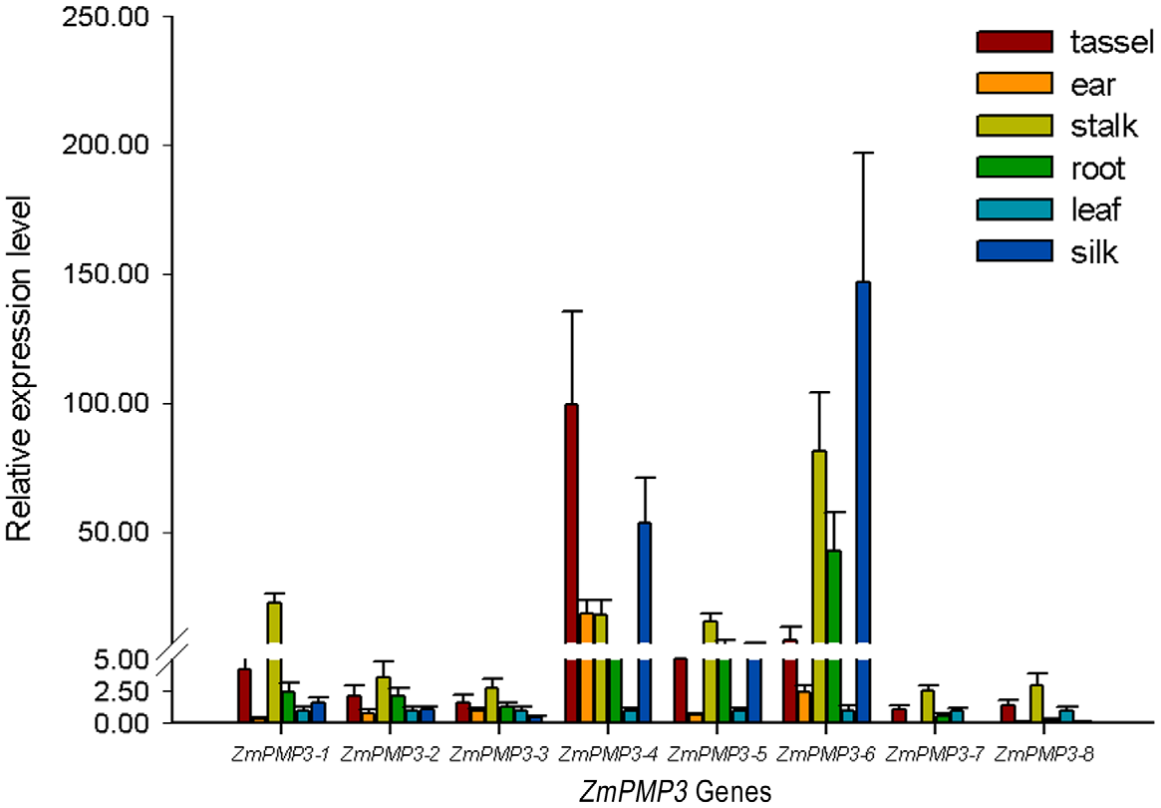
Expression patterns of the *ZmPMP3* genes in various tissues of maize. Total RNA was isolated from various tissues (mature leaves, mature roots, young silks, ears, tassels and stalks) at V12 and VT stage. Maize *GAPDH* gene was selected as internal control. Data from qRT-PCR experiments were analyzed according to the 2^−ΔΔCT^ method. Data represents the average of three independent experiments ±SE. All genes expression levels was assigned a value of 1 in leaf.

To investigate spatial expression patterns of the *ZmPMP3* genes in maize, different tissues including leaves, roots, ears, tassels and stalks were selected and detached from CN165 at V12 stage due to yield reduction caused by crop sensitivity to abiotic stresses [32]. Silks, as an important female tissue, are sensitive to abiotic stresses. Thus, silks at VT stage were also included in this experiment. The results showed that the transcript levels of the eight *ZmPMP3* genes were all at their peaks in the young organs and tissues. The transcript levels of *ZmPMP3-1*, *ZmPMP3-2*, *ZmPMP3-3*, *ZmPMP3-5*, *ZmPMP3-7* and *ZmPMP3-8* were detected at the highest levels in young stalks, and were detected at very low levels in leaves or roots (Figure 4). However, the *ZmPMP3-7* transcripts were detected at very low level in ears and silks. In addition, the transcripts of *ZmPMP3-4* and *ZmPMP3-6* were detected at the highest levels in tassels and silks, respectively. This result indicated that the activities of the different ZmPMP3 isoforms were confined to specific organs or tissues. Therefore, it was most likely that they functioned in different organs under normal or stress conditions.

### Functional complementation of *ZmPMP3s* in yeast mutant

The *PMP3* gene of *S. cerevisiae* encodes a homologue of the maize ZmPMP3s. Deletion of *PMP3* in yeast causes the hyperpolarization of cell membrane and consequently disrupted ion homeostasis. The corresponding mutant strain is sensitive to the toxic Na^+^ and hygromycin B [16]. Thus, function analysis of ZmPMP3s could be conducted by investigating the capacity of the *ZmPMP3* genes to complement the deletion of *PMP3* in yeast. The *ZmPMP3* genes with ADH1 promoter were strongly expressed in the YR93-31 (YR93-1 Δ*pmp3*) cells (the strains *ZmPMP3-1* to *ZmPMP3-8* genes over-expressed were named as YR93-31M1 to YR93-31M8, respectively). YR93-1 (*MATa ade2 his3*-Δ*200 leu2-3,112 lys2*-Δ*201 ura3-52 gal2* Δ*pmr2-2::HIS3* Δ*nha1::LEU2*) was used as a control.

**Figure 5:**
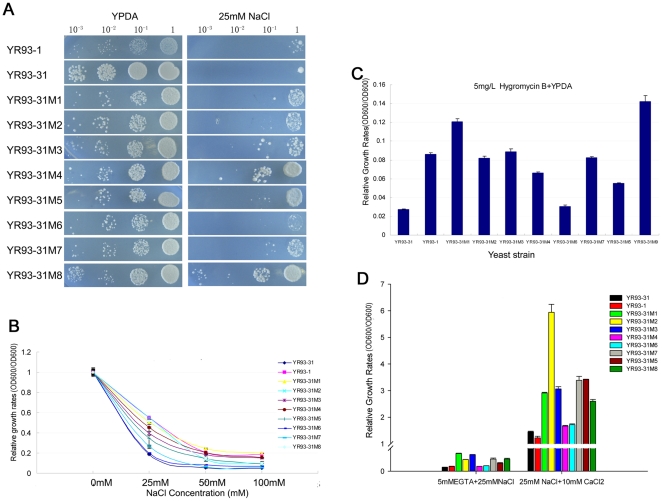
Functional complementation in yeast mutant strains. **A**) The 10-fold dilutions of YR31-1, YR93-31, and YR93-31M1-8 strains were spotted onto solid YPDA plates and YPDA supplemented with 25 mM NaCl. **B**) Dose- response growth curve of YR93-1, YR93-31, and YR93-31Ms (YR93-31 transferred by pAUR123-*ZmPMP3s*) strains of yeast under various concentrations of NaCl. **C**) The relative growth rates of YR93-1, YR93-31, and YR93-31M1-8 yeast strains under 5 mg/L hygromycin B treatment. **D**) The relative growth rates of YR93-1, YR93-31, and YR93-31M1-8 yeast strains under low calcium and high calcium conditions. Values are mean±SE (n = 3).

Both 10-fold dilution dot experiment and dose-response curves demonstrated that the expression of the *ZmPMP3* genes could suppress the sensitivity of mutant strains to the Na*^+^* and hygromycin B (Figure 5). As shown in Figure 5A and B, the salt sensitivity of YR93-31M1-5 and YR93-31M7-8 were restored. But the relative growth rate of YR93-31M6 was 18%, which was similar to YR93-31's. The salt sensitivity of YR93-31M7 was partly restored. Under 5 mg/L hygromycin B (Figure 5C) conditions, the sensitivity of the yeast YR93-31Ms to hygromycin B was reversed to the levels which were the same as that of the passive control YR93-1 under hygromycin B condition. YR93-31M6 also showed similar hygromycin B tolerance to YR93-31 strain. These results suggested that the ZmPMP3s abolished the hyperpolarization of cell membrane resulting from *PMP3* deletion and consequently maintained intracellular ion homeostasis.

Salt tolerance of yeast was reported to be affected by Ca^2+^ [9]. Therefore, we investigated the salt tolerance of YR93-31M1-8 under high and low calcium conditions (Figure 5D). On low calcium (YPDA+5 mM EGTA+25 mM NaCl), the salt sensitivity of all mutant strains YR93-31, YR93-1 and YR93-31M1-8 were exacerbated. However, the expression of the *ZmPMP3* genes still stimulated the growth of mutant strain YR93-31. On high calcium (YPDA+10 mM CaCl_2_+25 mM NaCl), the salt sensitivity of YR93-31 strain was reversed, the result being consistent with previous reports [16], [22]. Thus, under either high or low Ca^2+^ conditions, the expression of the *ZmPMP3* genes decreased the sensitivity of mutant trains YR93-31 to salt stress. These results suggested that although over-expression of the *ZmPMP3* genes in yeast enhanced the growth of yeast deletion mutant under salt stress, this process was not dependent on the presence of Ca^2+^.

### Salt tolerance of *ZmPMP3-1* overexpressed transgenic *Arabidopsis*

**Figure 6:**
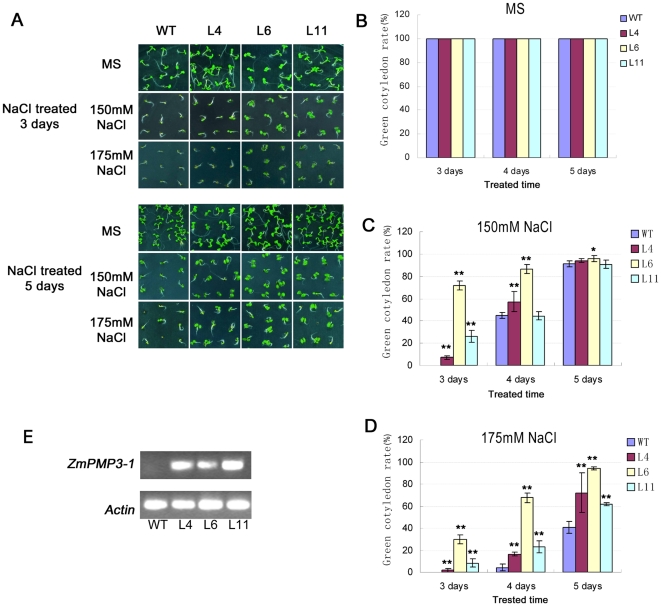
Comparison of green cotelydon rates between ZmPMP3-1 transgenic plants and WT. **A**) The performance of transgenic lines and WT in MS plate and 150 mM 175 mM NaCl plates. The L4, L6, L11 represent three different independent transgenic lines. **B**, **C**, and **D**) Comparison of the green cotyledon rates between transgenic lines (L4, L6 and L11) and WT. The green cotelydon rates were calculated 5-days after sowing under normal, 150 mM and 175 mM NaCl treatments. The number of plants is expressed as means SEM (n = 40 plants). A statistic difference measurement from three independent replicates. “*” indicates significant difference between transgenic plants and WT control with F-test (* P<0.05, ** P<0.01). **E**) Transcript level of *ZmPMP3-1* in transgenic plants (L4, L6, and L11) and WT. Total RNA was extracted from leaves of 3-week old plants grown under normal conditions. The transcript level of *ZmPMP3-1* was measured by RT-PCR. Thirty PCR cycles were used, and the Arabidopsis *Actin2* gene was amplified as the control.

*ZmPMP3-1* was selected to investigate whether the *ZmPMP3* genes could improve salt tolerance of plants because it was responsive to various abiotic stresses and encoded protein showed higher identity to *AtRCI2* genes. The full-length ORF of *ZmPMP3-1* controlled by CaMV35S promoter was transferred into Columbia-0 type *Arabidopsis*. Of 35 individual T1 transgenic plants generated, 20 were positive transformants. Co-segregation analysis of T2 generation showed that 8 of 20 individuals had one copy T-DNA insert. Three independent transgenic lines, i.e. L4, L6 and L11, with the similar performances, were used in phenotypic analysis of transgenic plants. These positive transgenic and wild type plants were chosen for testing with three concentrations of NaCl (0 mM, 150 mM and 175 mM). Under normal conditions, all of the transgenic lines showed similar performances comparing with the wild type (WT). Germination rates of the transgenic lines and the WT plants did not show significant difference under salt stress (data not shown). However, the WT showed delayed post-germination growth comparing with the transgenic lines under salt condition (Figure 6). As shown in Figure 6A, B and C, under 150 mM NaCl treatment, 3%∼29.4% of the transgenic plants had two cotyledons at the third day after sowing, while the WT plants had no cotyledons. At the fifth day, there was no significant difference between the transgenic lines and the WT. Under the 175 mM NaCl (Figure 6A, B and D), the performance was similar to what happened under 150 mM NaCl condition. The transcripts of *ZmPMP3-1* were investigated in transgenic lines L4, L6, and L11 (Figure 6E).

**Figure 7:**
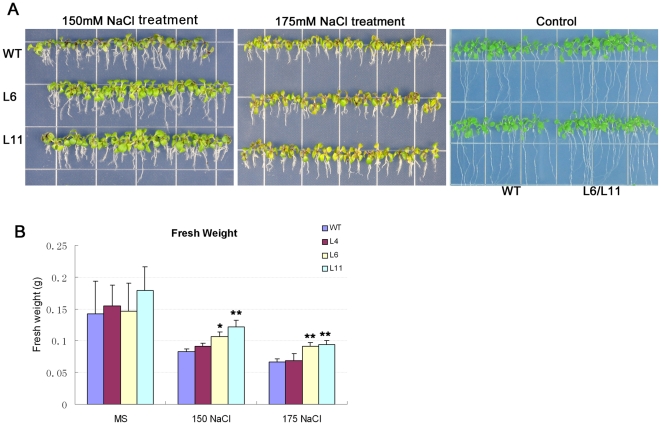
Comparison of fresh weight between *ZmPMP3-1* transgenic plants and WT. **A**) The performance of transgenic lines (L4, L6, and L11) and WT in 150 mM, 175 mM NaCl plates and control. The photos were taken two-week after sowing. **B**) Comparison of fresh weights between transgenic lines (L4, L6 and L11) and WT. The fresh weights were calculated two-week after sowing. The number of plants is expressed as means SEM (n = 20 plants). A statistic difference measurement from three independent replicates. “*” indicates significant difference between transgenic plants and WT. F-test (* P<0.05, ** P<0.01).

**Figure 8:**
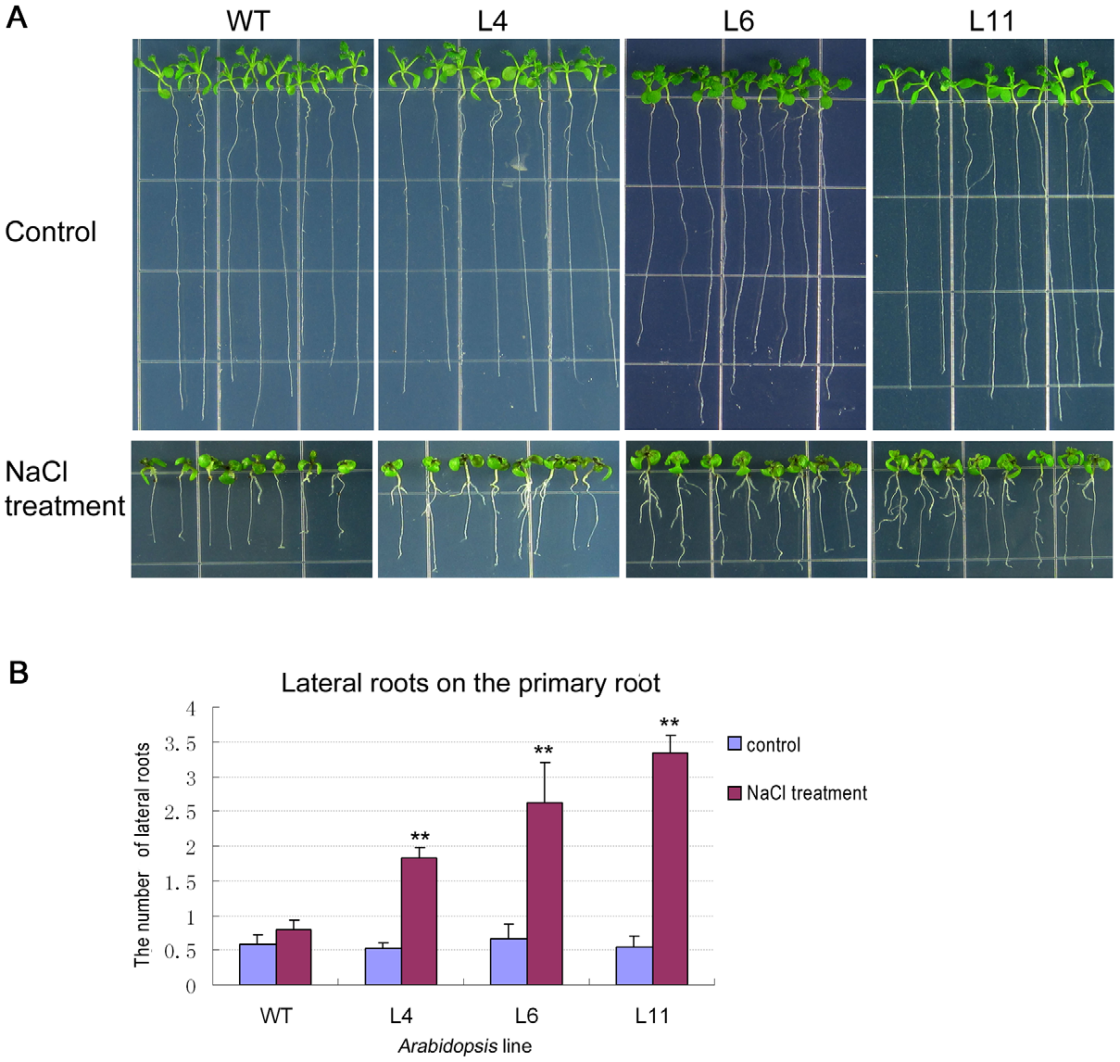
Comparison of the number of lateral roots between *ZmPMP3-1* transgenic plants and WT. **A**) The root growth performance of transgenic lines (L4, L6 and L11) and WT in MS and 200 mM NaCl plates. The photos were taken 10-day after transferring. **B**) Comparison of the lateral root number between transgenic lines (L4, L6, and L11) and WT. The 0ne-week old seedlings were transferred to MS and 200 mM NaCl plates. The numbers of lateral roots were determined 10-day after transferring. The number of plants is expressed as means SEM (n = 10 plants). A statistic difference measurement from three independent replicates. “*” indicates significant difference between transgenic plants and WT. F-test (* P<0.05, ** P<0.01).

Furthermore, the fresh weights of two week old seedlings sowed on MS media containing 150 mM and 175 mM NaCl were monitored (Figure 7A, B). The fresh weights of L6, and L11 lines were significantly higher than the fresh weight of the WT. To investigate the phenotype of roots, the transgenic and the WT seedlings were transferred to the MS and 200 mM NaCl plates (Figure 8). Under normal condition, there was no difference observed between the transgenic and the WT seedlings. However, under salt stress condition, the numbers of lateral roots of the transgenic lines were significantly larger than the WT. These results clearly revealed that overexpression of *ZmPMP3-1* improved salt tolerance of transgenic *Arabidopsis* plants and eliminated growth retardation caused by salt stress.

**Figure 9:**
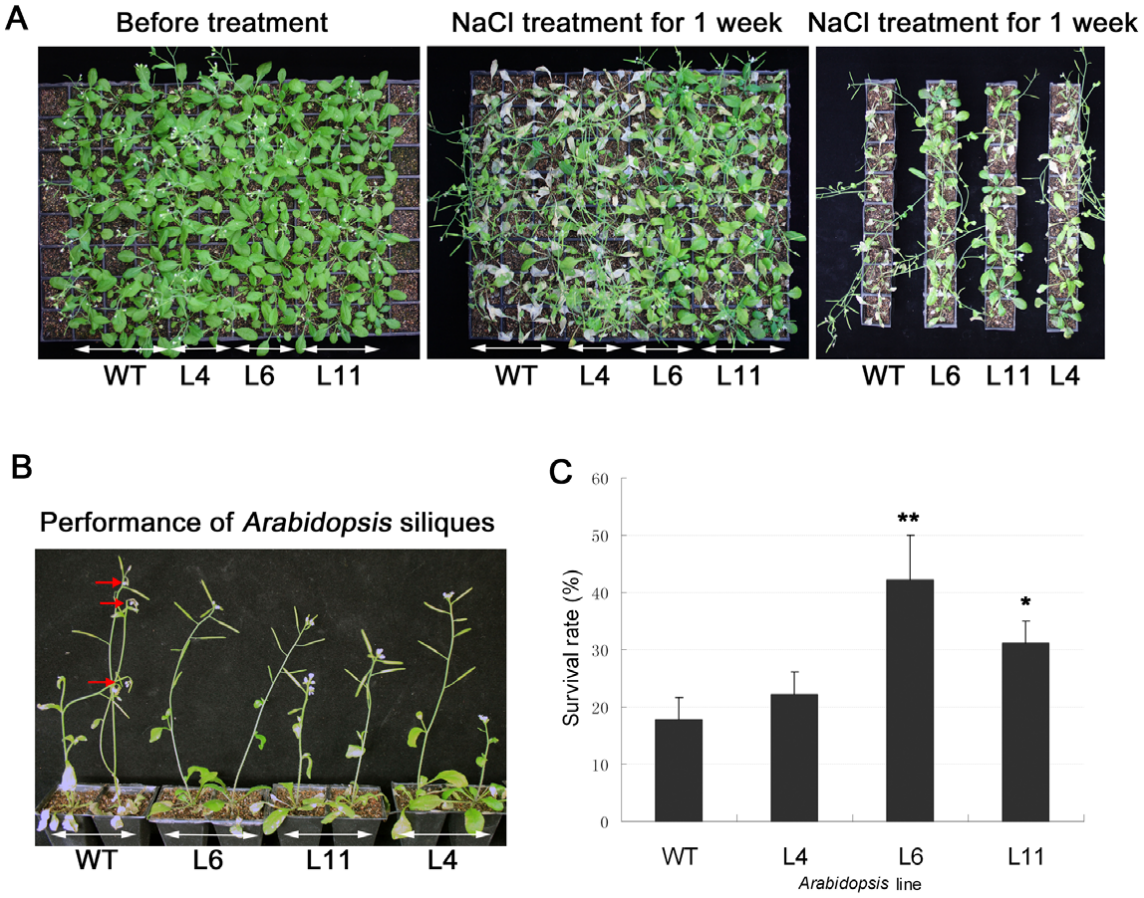
Analysis of the survival rate in *ZmPMP3-1* transgenic plants and WT. **A**) The performance of *ZmPMP3-1* transgenic lines (L4, L6 and L11) and WT under salt condition and control. The 4 week-old transgenic and WT plants were irrigated by the 300 mM NaCl solution for one week. Here the transgenic plants grew well, but the WT plants exhibited chlorosis and sterility. **B**) The performance of the anthotaxy tips of transgenic lines (L4, L6 and L11) and WT under salt stress. After one week salt treatment, the almost anthotaxy tips of WT plants exhibited chlorosis. The red arrows marked the wilted anthotaxy tips of WT. **C**) comparison of the survival rate between transgenic lines and WT. Each value represents the average of 15 plants with three replicates. Values are mean±SE. “*” indicates significant difference between transgenic plants and WT. F-test (* P<0.05, ** P<0.01).

Plants at silique stage were irrigated by 300 mM NaCl solution to investigate salt tolerance of mature plants. After one week, only ∼18% of the wild type plants survived, while 22%–42% of the transgenic plants survived. Under normal condition, the transgenic and the WT plants all grew well (Figure 9A, C). As it shown in Figure 9B, most of the transgenic lines continued to grow well at the presence of the 300 mM NaCl, whereas almost anthotaxy tips of the WT plants exhibited chlorosis and sterility after 1-week NaCl treatment. This result indicated that overexpression of *ZmPMP3-1* conferred salt tolerance to the transgenic plants at reproductive stage.

### Photosynthetic rate and CMS of *ZmPMP3-1* overexpressed transgenic *Arabidopsis*

**Figure 10:**
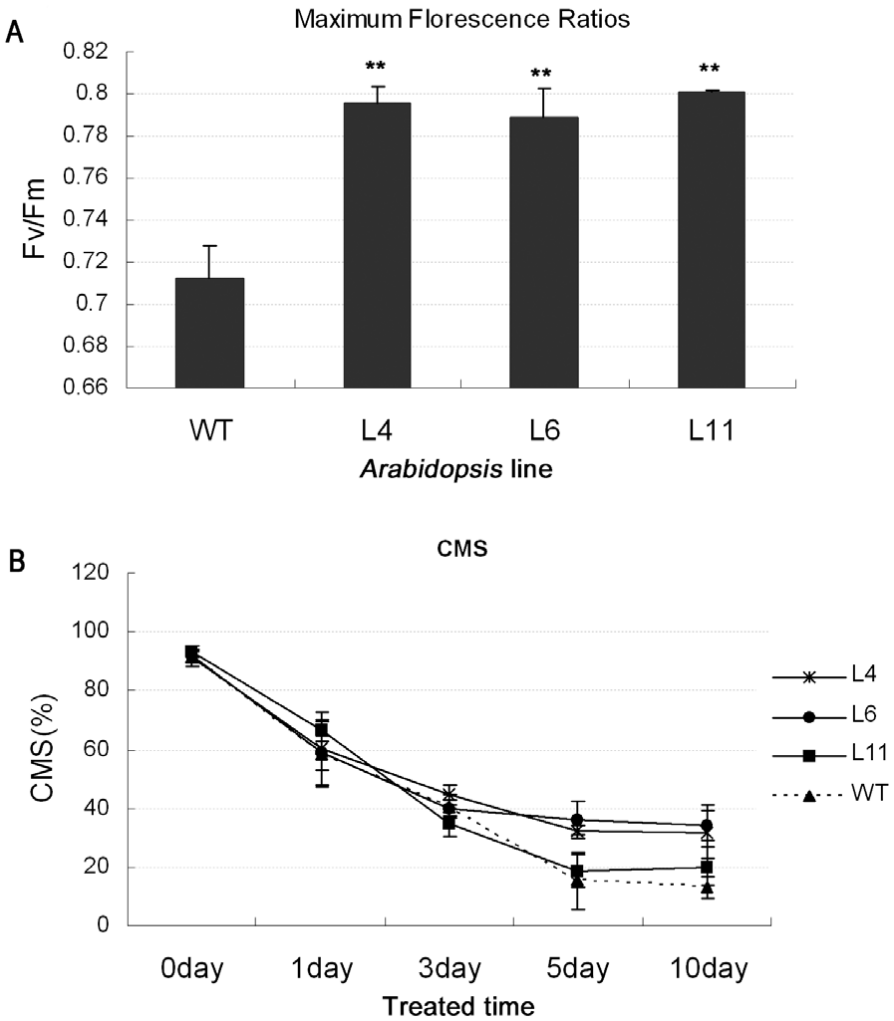
Elucidation of the CMS and Photosynthetic rate of *ZmPMP3-1* transgenic plants. **A**) Comparison of maximum fluorescence ratios (Fv/Fm) between transgenic lines (L4, L6 and L11) and WT. The Fv and Fm values were measured to calculate the maximum fluorescence ratios (Fv/Fm). The Fv and Fm values of 4 week old *Arabidopsis* were measured after one week treatment by 300 mM NaCl. The number of plants is expressed as means SEM (n = 8 plants). A statistic difference measurement from three independent replicates. “*” indicates significant difference between transgenic plants and WT. F-test (* P<0.05, ** P<0.01). **B**) Comparison of the CMS between transgenic lines (L4, L6 and L11) and WT. The three-old plants were treated with 300 mM NaCl and the CMSs were determined at selected time points. The CMS values represented the average of six individual plants.

During the period of increased salt stress, plants often exhibit stress symptom, including increased leaf senescence and cellular damage due to the photo-oxidative stress [33]–[37]. The chlorophyll florescence and CMS (cell membrane stability) as important physiological probes were determined to investigate effects of salt stress on plants. To determine the chlorophyll florescence, the 4-week old *Arabidopsis* plants were treated for one week by 300 mM NaCl and then Fv and Fm values were measured to calculate the maximum florescence ratios (Fv/Fm), which represents the photosynthetic rate of plants. The results showed that the Fv/Fm values of the transgenic plants were significantly higher than the WT, indicating that the chlorophyll of the WT was damaged more easily by salt stress (Figure 10A). The difference of the CMS between the transgenic and the WT plants was observed until the fifth day, and the CMS of the transgenic lines was significantly higher than the WT (Figure 10B). Taken above, these results indicated that the transgenic lines could be less damaged than the WT under high salinity environment.

### Expression patterns of stress- and ion transporter-related genes in *ZmPMP3-1* over-expressed transgenic plants

To understand the molecular mechanism underlying the enhanced salt tolerance by 35S-*ZmPMP3-1*, we tested transcript levels of a set of selected abiotic stress-responsive genes and antioxidants genes in the transgenic and WT plants. Because transgenic yeasts reestablish ion homeostasis under high salt conditions, several genes modulating ion homeostasis were also selected (Table 2, S1). Under salt conditions, several stress-responsive genes including the stress-response gene *DREB2A* and its target gene *RD29B* were up-regulated more highly in the transgenic plants than in the WT. Expression of an ABA-response gene *RAB18* was also elevated (Table 2). The ion transporter-related genes, including Na^+^/H^+^- antiporter gene *NHX1*, and H-ATPase genes *AVP1*, and *AHA2*, were up-regulated in the transgenic plants than in the WT. The up-regulation of these genes in the transgenic plants suggested that over-expressing *ZmPMP3-1* affected the expression of the ion transport-related genes. In addition, several antioxidant genes, such as *P5CS1-2*, *CAT1*, *CCS*, and *CSD1-2*, exhibited up-regulation in the transgenic plants (Table 2). Under normal conditions, the transcript levels of all genes were similar between the transgenic and WT plants.

**Table 2:** Up-regulated genes in *ZmPMP3-1* overexpressed transgenic plants.

Name^a^	Accession NO.^b^	Function annotation^c^	Relative expression1^d^	Relative expression2^e^
*CSD1*	At1g08830	Superoxide dismutase1	1.106±0.192	2.162±0.315
*CSD2*	At2g28190	Superoxide dismutase2	1.453±0.044	2.037±0.053
*P5CS1*	At2g39800	Delta1-Pyrroline-5-Carboxylate synthase 1	1.185±0.153	3.252±0.470
*P5CS2*	At3g55610	Delta1-Pyrroline-5-Carboxylate synthase2	0.735±0.018	3.110±0.697
*CCS*	At1g12520	Copper Chaperone for SOD1	1.179±0.057	2.645±0.032
*CAT1*	At1g20630	Catalase 1	1.186±0.049	2.440±0.067
*RD29B*	At5g52300	Responsive to Dessication 29B	1.373±0.086	3.529±0.232
*DREB2A*	At5g05410	DNA binding/transcription activator/transcription factor	1.364±0.097	5.159±0.843
*RAB18*	At1g43890	Responsive to ABA 18	2.389±0.086	3.473±0.418
*AVP1*	At1g15690	ATPase/hydrogen-translocating pyrophosphatase	0.939±0.186	2.149±0.201
*NHX1*	At5g27150	Na^+^/H^+^ antiporter	0.889±0.028	5.861±0.504
*AHA2*	At4g30190	ATPase/hydrogen-exporting ATPase	1.138±0.115	5.406±0.522

a Name of the selected genes.

b Accession number of the selected genes in GenBank.

c Function annotation of the selected genes in GenBank.

d Comparison of the relative expression level of selected genes in *ZmPMP3-1* transgenic plants and WT under normal conditions.

e Comparison of the relative expression level of selected genes in *ZmPMP3-1* transgenic plants and WT under salt stress conditions.

The 4 week old *ZmPMP3-1* overexpressed transgenic *Arabidopsis* plants were harvested after 3 days treated by 300 mM NaCl. The transgenic line and WT were used in transcript level analysis of selected genes. Quantitative real-time PCR was performed and data was analyzed by the 2^−ΔΔCT^ method. When the gene expression fold of a given gene ≥2 (P value<0.05), the gene is regarded as up-regulated. *Actin* of Arabidopsis was used as an internal control.

## Discussion

### *ZmPMP3* genes are conserved in sequence, but different in responses to abiotic stresses

The *PMP3*-type genes have been described in several plants, including *Arabidopsis*, rice, wheat, barley, sheep grass, alkali grass, and plaintan [17]–[22], [38]. Eight in *Arabidopsis* (*AtRCI2A-H*) and twelve in rice (*OsRCI2-1* to *OsRCI2-12*) members have been reported, respectively [23]. But only the *OsRCI2-3*, *OsRCI2-5*, *OsRCI2-6* (*Oslti6B*), *OsRCI2-8*, *OsRCI2-9, OsRCI2-10* (*Oslti6A*) and *OsRCI2-11* have the corresponding cDNA sequences in rice (based on searching results in RGAP: http://rice.plantbiology.msu.edu/). Additionally, the PMP3-type proteins are also present in metazoans and prokaryotes, but not in animals such as arthropods and vertebrates [23]. The ZmPMP3s sequences obtained in this study showed high identity to AtRCI2s and OsRCI2s. The eight ZmPMP3s were divided into three groups according to their amino acid sequences. The conserved amino acid sequences and subcellular localization indicated that they shared conservative functions as membrane proteins. However, the *ZmPMP3* genes were responsive differently to abiotic stresses and ABA.

The expression of *ZmPMP3* homologues, such as *Blt101* in barely, *wpi6* in wheat, *Oslti6a* and *Oslti6b* in rice, *AcPMP3-1 and AcPMP3-2* in sheep grass, accumulated under salt, drought, cold, and ABA conditions [15], [21], [39], indicating their roles in abiotic stresses tolerance. In this study, *ZmPMP3s* were mainly responsive to salt and drought stresses. Only four genes were responsive to cold stress. Under salt and drought stresses, the inductions or repressions of the *ZmPMP3* genes were dependent on stress duration. The rapid and transient rise of the transcripts of *ZmPMP3-1*, *ZmPMP3-5* and *ZmPMP3-6* suggested that these ZmPMP3s mediate an early protection against sudden abiotic stresses before an overall and permanent adaption. The stronger inductions of *ZmPMP3-5* and *ZmPMP3-6* were observed only in root, indicating their organ specific expression in response to abiotic stresses at seedling stage. The differences of expression patterns among the eight genes under abiotic stresses implied that although the genes are conserved structurally, their regulation in response to abiotic stresses is perhaps altered due to specific adaptations to environmental conditions.

Under abiotic stresses, plants may produce hormones such as ABA which in turn causes stomatal closure and induces expression of stress-related genes. Some genes are induced by extracellular ABA treatment, whereas others are not [40]. In this study, four of the eight members of these *ZmPMP3* genes, including *ZmPMP3-2*, *ZmPMP3-4*, *ZmPMP3-5* and *ZmPMP3-6*, were regulated by ABA, and the others were not. This result showed that these genes were differently responsive to ABA, suggesting that they were involved in different signaling pathways. The *ZmPMP3-2*, *ZmPMP3-4*, *ZmPMP3-5* and *ZmPMP3-6* may be involved in ABA-dependent signal pathway, and the others were not.

Additionally, the transcript levels of *ZmPMP3s* showed different degrees in various organs. The transcripts of *ZmPMP3-1*, *ZmPMP3-2*, *ZmPMP3-3*, *ZmPMP3-5*, *ZmPMP3-7* and *ZmPMP3-8* were accumulated mainly in stalks, and moderately in tassels. *ZmPMP3-4* and *ZmPMP3-6* were accumulated mainly in tassels and silks, respectively. These results indicated that in spite of their high sequence similarity and conserved subcellular localization, the eight ZmPMP3s did not have the exactly same function.

### ZmPMP3s have a common function of maintaining ion homeostasis and this process is not dependent on the presence of Ca^2+^

The Pmp3p in yeast, a homologue of ZmPMP3s, contributes to the regulation of intercellular ion homeostasis through controlling the plasma membrane potential, which prevents excessive build-up of the Na^+^ influx [16]. When PMP3 was absent, YR93-31 showed membrane hyperpolarization, and which in turn lead to high sensitivity to toxic ions, such as Na^+^ and hygromycin B [16]. Hygromycin B is a lipophilic toxic cation, which is taken up according to the membrane potential [41]. Recent research found that PMP3-type proteins, including AtRCI2A, Oslti6a/b, AcPMP3-1, and PutPMP3-1/2, have the capability of complementing the function of PMP3 lacking mutant yeast strain and restore salt sensitivity of this lose-function strain [16], [22]. It is suggested that these PMP3-type proteins were most likely to be involved in regulation of membrane potential and consequently regulated the ion homeostasis under ionic stress condition. The complementation analysis in this study revealed that ZmPMP3s, except ZmPMP3-6, could restore salt and hygromycin B sensitivity of YR93-31 strain. It is suggested that ZmPMP3s have a common function of regulating membrane potential, consequently maintaining intracellular ion homeostasis.

The calcium ion (Ca^2+^) is firmly established as a ubiquitous intracellular second messenger in plants, which acts not only the regulator of intercellular osmotic potential, but also as an important signal factor in stress signal transduction pathway in plants, such as Ca^2+^-CaM signal pathway [42], Ca^2+^-CDPK signal pathway [43], and SOS system [44]. All of these pathways are linked to the downstream cell response to abiotic stresses. It is reported that the yeast has a similar stress-related Ca^2+^-signaling pathway to plants [9]. Our research showed that the salt sensitivity phenotype of YR93-31 was relieved in the presence of Ca^2+^. It is possible that Ca^2+^ activates a Ca^2+^- and calmodulin-dependent protein phosphatase (CaN), which promotes the expression of stress-related genes, and then confers high salt tolerance to yeasts [45]. Alternatively, there is a class of non-selected ion channel like NSC1 (non-selected ion channel 1) that is sensitive to Ca^2+^, which could inhibit Na^+^ uptake in the presence of millimolar Ca^2+^ [46]. However, the function of ZmPMP3s seems to be independent on the presence of Ca^2+^. Under either high calcium or low calcium conditions, YR93-31Ms expressing *ZmPMP3s* still showed lower salt sensitivity than YR93-31 strain. Therefore, it suggested that the process of ZmPMP3s regulating membrane potential was independent on the presence of Ca^2+^.

### Physiological and molecular mechanisms of *ZmPMP3-1* which enhanced salt tolerance

Salt stress has three different effects: it reduces water potential, causes ion imbalance and toxicity, and then results in a clear stunting of plants [47]. In this study, *ZmPMP3-1* over-expression in *Arabidopsis* relieved growth inhibition caused by salt stress, as evident from green cotyledon rate, biomass and root morphology of the transgenic plans at seedling stage.

Physiological indices, including Fv/Fm and CMS [36], [37], are typical physiological parameters for evaluating abiotic stresses tolerance and resistance in crop plants. The maximum efficiency of PSII photochemistry, measured as Fv/Fm, is a direct reflection of the PSII activity, and environmental stresses are associated with decreased Fv/Fm ratio. In this study, the transgenic plants under salt condition exhibited lower decline of photosynthetic rate (Fv/Fm). Thus *ZmPMP3-1* plants might have more robust photosynthetic capabilities than the WT. CMS determinations were consistent with Fv/Fm results and suggested that the transgenic plants had enhanced salt-stress tolerance.

Ion cytotoxicity is caused by replacement of K^+^ by Na^+^ in biochemical reactions and conformal changes and loss of function of proteins as Na^+^ interfere with noncovalent interaction between their amino acids. Metabolic imbalance caused by ionic toxicity may also lead to oxidative stress [44]. Plants possess a number of antioxidants that protect against the potentially cytotoxic species of activated oxygen, such as superoxide dismutase (SOD), catalase (CAT), guaicol peroxidase (POD), and so on. The plants with higher activation of antioxidants have been reported to show higher salt tolerance [48]–[50]. In this study, the transcripts of *P5CS1-2*, *CAT1*, *CCS*, and *CSD1-2* were more highly accumulated in the transgenic plants than in the WT. The *CDS*, *CAT* and *CCS* genes encode superoxide dismutase (SOD), catalase (CAT), and copper chaperone for SOD1, respectively, which are important antioxidants in ROS scavenging system. *P5CS1-2* genes encode delta-1-pyrroline-5- carboxylate synthase, which plays a key role in biosynthesis of proline. Transgenic plants overexpressing these genes always show higher tolerance of salt stress than non-transformed control [51], [52]. Thus the enhanced salt tolerance of transgenic plants might be attributed to the up-regulation of these antioxidants genes.

Theoretically, three mechanisms can be explained for ion homeostasis in plant symplast. They are respectively, (i) preventing the Na^+^ entry from cell membrane; (ii) compartmenting the excessive Na^+^ into vacuoles; and (iii) exporting Na^+^ back to external medium [53]. In *Arabidopsis thaliana*, the function of *NHX1* and *AVP1* genes in maintenance ion homeostasis were well established [54]–[57]. AtNHX1 has the activity of Na^+^/H^+^-antiporter, it might compartmentalize the excessive Na^+^ into vacuole [56]. *AtAVP1* encodes P-ATPase, which participates indirectly in the maintenance of ion homeostasis by generating the H^+^ gradient [57]. Brini *et al.* found that co-expression of wheat *NHX1* and *AVP1* could improve salt tolerance of transgenic *Arabidopsis* by compartmentalizing excessive Na^+^ into vacuole [58]. In this study, the accumulation of *AtNHX1*and *AtAVP1* transcripts was significantly higher in the transgenic lines than in WT under salt conditions. The yeast functional complementation showed that ZmPMP3s had the function of controlling membrane potential. In fact, previous research on guard cells by voltage-clamp demonstrated that the main transporters in membrane were responsive to membrane voltage [59]. Thus, it is speculated that *ZmPMP3-1* affects the expression of some ion transporter genes, including *NHX1* and *AVP1*, by regulating membrane potential, and consequently affect intracellular ion homeostasis. In further research, more efforts should be given to the deciphering the signals and molecular mechanism of *ZmPMP3* in regulating ion homeostasis.

The present study identified eight *ZmPMP3* genes in maize. Possibly, these genes share a common function of cell membrane depolarization, and consequently maintain intercellular ion homeostasis. The functions of these genes were similar and related, but not exactly the same. These eight genes were responsive to abiotic stresses differently, and accumulated mainly in different organs. The investigation of molecular mechanism in *ZmPMP3-1* over-expressed transgenic plants could aid in the elucidation of the regulatory mechanism of PMP3 in response to abiotic stresses and maintenance of ion homeostasis in plants.

## Materials and Methods

### Plant material, grown conditions, and stress treatment

A maize inbred line “CN165” was used in this study, which is the female parent of commercial maize hybrid “Zhongyu 4” and have strong stress tolerance under poor soil condition during the vegetative and reproductive stages.

For salt and drought stress, the maize seedlings at V3 stage in green house (28°C, humidity 40–50%, ∼150 µmol/m^−2^ s^−1^ under a 12-h light/12-h darkness photocycle) were transferred into 200 mM NaCl and 30% PEG-6000 solution and then harvested at given time points. For cold stress, the maize seedlings at V3 stage were transferred into a 4°C chamber and harvested at given time points. For ABA treatment, maize seedlings at V3 stage were sprayed with 100 µM ABA solution. Shoots and roots which were sampled respectively from the seedlings, were frozen immediately with liquid nitrogen, and stored in −80°C for RNA isolation and real-time quantitative PCR (qRT-PCR) analysis.

To investigate the spatial expression patterns of the *ZmPMP3s* in maize, different tissues were selected and detached from CN165 at V12 stage, including leaf, root, ear, tassel and stalk. In addition, the silk at VT stage was included in this analysis. The maize plants were well irrigated (volumetric water content 22%–40%, TDR300 Soil Moisture Meter produced by Spectrum Technologies. Inc) during sampling and five individual plants were used in tissue sampling. Tissues and organs were frozen immediately with liquid nitrogen, and stored in −80°C for RNA isolation and qRT-PCR analysis.

*Arabidopsis thaliana* ecotype Columbia (Col) was used as WT and phenotypic assays of *35S-ZmPMP3-1* plants in all experiments. Seeds were vernalized for 3 days in 4°C on MS (Murashige and Skoog) medium plates, and then cultured in green house (22°C, humidity 40–50%, 120–150 µmol/m^−2^ s^−1^ under 16-h light/8-h darkness). The seedlings grown for 7 days on MS plates were transferred to the soil (nutrition earth: vermiculite = 1∶1) and cultured for 3 weeks. The 3-week old wild type and transgenic *Arabidopsis* plants were irrigated with 300 mM NaCl solution for 3 days, and then harvested. These plants were frozen immediately with liquid nitrogen for qRT-PCR analysis.

### Isolation and bioinformatic analysis of sequences of *ZmPMP3* genes

Based on an EST homologous to the plasma membrane protein 3 (Pmp3p) which was obtained from a previously constructed cDNA library [60], the specific full-length primers were designed as follows: F, 5′-AGCGAAAGGAGAGAAGGAATC-3′ and R, 5′-GATGGGGTGGGTACGGTAG-3′. The full-length cDNA fragment was obtained in CN165 by rapid amplification of cDNA 3′ end. The conditions for amplification were 95°C for 5 min, then 30 cycles at 95°C for 30 s, 58°C for 30 s, and 72°C for 1 min, followed by 72°C for 10 min. The cDNA full-length fragment was purified, sequenced, and named as *ZmPMP3-1*.

To explore other *ZmPMP3*-related genes in maize, blastx and blastn were conducted by comparing the sequences of *ZmPMP3-1* and homologous genes such as *AtRCI2a*, *AtRCI2b*, *Oslti6a*, *Oslti6b*, *AcPMP3-1*, *AcPMP3-2*, *Blt101*, *PutPMP3-1*, and *PutPMP3-2*, with the entries in NCBI GenBank (http://www.ncbi.nlm.nih.gov). Seven putative protein sequences with E-value≤10^−6^ and their corresponding full-length cDNA sequences were obtained. The specific primers were designed based on the seven corresponding cDNA sequences (Table S2). Full-length cDNA fragments were amplified from salt, cold and drought-induce first-strand cDNA by RT-PCR (Dataset S1). Amplification and sequencing of the full-length cDNA fragments were repeated three times. The calculation of the putative protein sequences was carried out by NCBI ORF finder (http://www.ncbi.nlm.nih.gov/gorf/gorf.html) (Dataset S2) and the conserved domains were found by blast in the PROSITE database (http://expasy.org/prosite/) [61]. The prediction of the transmembrane helices of these putative protein sequences was executed by the TMHMM 2.0 tool on line (http://www.cbs.dtu.dk/services/TMHMM/). The ProtScale tool (http://web.expasy.org/protscale/) was used to analyze hydrophobic domains (Figure S1) [62]. Phylogenetic tree based on the resultant alignments was constructed by using the neighbor-joining method with the MEGA 4 program [63], [64]. The genomic sequences of *ZmPMP3* genes were PCR-amplified from genomic DNA of the inbred CN165 using specific primers (Table S2 & Dataset S3).

### Subcellular localization of ZmPMP3s-GFP fusion proteins

The ORF (open reading frame) sequences without terminating codons of the *ZmPMP3* genes were cloned into *Xba*I/*Sca*II sites of pBI221-GFP vector [31], to generate *ZmPMP3-GFP* fusion genes driving by CaMV 35S promoter. The specific primers containing *Xba*I and *Sca*II were shown in Table S3. The resulting constructs were transferred into onion epidermal cells by particle bombardment with gene gun (Biorad Helios™). The pBI221-GFP vector was used as control. After incubation on MS plate for 16–24 hours, the onion epidermal cells were observed with a laser scanning confocal microscope (Leica TCS-NT). For imaging the fluorescence of GFP, the excitation line was 488 nm for GFP, and GFP signal was collected under 465–490 nm bandwidths.

### Complementation experiment of the *ZmPMP3* genes

The yeast mutant strain YR93-31 (YR93-1 Δ*pmp3*), lacking yeast PMP3, was used to test whether the *ZmPMP3* genes could functionally replace yeast PMP3. The strain YR93-1 (*MATa ade2 his3*-Δ*200 leu2-3,112 lys2*-Δ*201 ura3-52 gal2* Δ*pmr2-2::HIS3* Δ*nha1::LEU2*) was used as control. The YR93-1 strain is sensitive to salt comparing with the wild type YR93, because the deletion of both plasma membrane Na^+^/H^+^-antiporter (NHA1) and Na^+^-ATPase (PMR2) leads to the disruption of these high affinity Na^+^ efflux system [16]. To examine the relationship between maintaining ion homeostasis and the function of *ZmPMP3* genes, the ORFs of the *ZmPMP3* genes were cloned into the pAUR123 vector (Takara, Japan) driven by the ADH1 promoter. The reconstructed vectors were transferred into yeast mutant YR93-31 (Table S4), and the resulting strains were named as YR93-31M1-8.

The YR93-1, YR93-31 and YR93-31M1-8 strains were cultured in YPDA liquid medium to an OD600-0.3, and then 20 µl of culture was transferred into 2 ml fresh YPDA liquid medium containing various concentrations of NaCl and 5 mg/L hygromycin B. Yeast was grown for 15 h at 30°C, and then absorbance (OD600) was measured. The absorbance (OD600) of culture under normal condition (without additional NaCl) was used as control. The values of relative growth rate were calculated by use of the formula: the relative growth rate = absorbance (OD600) of culture under NaCl or hygromycin B conditions/absorbance (OD600) of culture under control.

The 10-fold dilutions of YR31-1, YR93-31 and YR93-31M strains were spotted onto solid YPDA plates and YPDA supplemented with 25 mM NaCl. The strains were cultured overnight to an OD600-1.0, and then diluted to OD600-0.3. The diluted yeast strains were dotted onto solid YPD plates containing 2% agar. The plates were cultured for 2–4 days at 30°C, and then photographed.

The relative growth rates of YR93-1, YR93-31 and YR93-31M1-8 yeast strains on low calcium and high calcium. The histogram showed the values of relative growth rate of low calcium and high calcium under salt conditions. The absorbance (OD600) of culture in YPDA with 25 mM NaCl was used as control. The values were calculated by use of the formula: relative growth rate = absorbance (OD600) of culture under low calcium or high calcium conditions/absorbance (OD600) of culture under control. The values represented the averages of three replications.

### Generation of transgenic plants

The ORF of *ZmPMP3-1* was cloned into *Nco*I/*Bst*II sites of pCAMBIA3301 vector replacing the *GUS* gene, and *ZmPMP3-1* was controlled by the CaMV35S promoter. The primers with *Nco*I and *Bst*II sites were designed as follows: F-Nco, 5′-CATCGGCACCATGGCGGAGGGGACT-3′, R-Bst, 5′-ATGGGTCACCGTCGGCAAGGATGAC-3′. The reconstructed vector was transferred into *Agrobacterium* GV3101 and then transferred into wild type *Arabidopsis* (Columbia 0 ecotype) plants by *Agrobacterium tumaefaciens* mediated transformation. Positive transgenic plants over-expressing *ZmPMP3-1* were firstly screened on MS plates containing basta. T4 homozygous plants were used for further analysis.

### Phenotyping of stress tolerance and physiological parameters

Seeds were vernalized for 3 days in 4°C on MS plates, 150 mM and 175 mM NaCl plates, then cultured for 3, 5 and 14 days in green house (22°C, humidity 40–50%, 120–150 µmol/m^−2^ s^−1^ under 16-h light and 8-h darkness). Green cotyledons rate and fresh weights of these plants were evaluated during different periods. Seedlings grown 7 days on MS plate were transferred to vertical MS and 200 mM NaCl plates for 10 days, and then the numbers of lateral roots morphology were monitored. To characterize salt tolerance of mature plants, 7 day old seedlings grown on MS plate were transferred to the soil (nutrition earth: vermiculite = 1∶1) and cultured in green house. Plants at silique stage were irrigated with 300 mM NaCl solution for 7 days. Survival rates of these plants were evaluated and pictures were taken. Each value of green cotyledons rate represented the average of 40 seeds with at least three replicates. Each fresh weight was calculated based on the average weight of 20 individual plants with three replicates and the numbers of lateral roots were results from the averages of 8–10 plants with three replicates.

Plant cell membrane stability (CMS) was determined with a conductivity meter (DDS-1, YSI) and formula CMS (%) = (1−initial electrical conductivity/electrical conductivity after boiling)×100. Four-old-week *Arabidopsis* plants irrigated with 300 mM NaCl for each line were selected to determine CMS and the values were calculated based on the averages of six individual plants with two replicates [65]. Chlorophyll florescence was measured with OS-30p Chlorophyll Fluorometer (OPTI-SCIENCE, USA) according to the protocol, and maximum efficiencies of PSII photochemistry, Fv/Fm = (Fm−F0)/Fm, were used to assess changes in primary photochemical reactions of photosynthetic potential. Four-week-old *Arabidopsis* plants irrigated with 300 mM NaCl were chosen to collect Fv/Fm data. Fv/Fm value of each line represents average of eight individual plants with three replicates.

### Real time RT-PCR analysis

Total RNAs were isolated from maize and *Arabidopsis* plants by TRIZOL reagent (Invitrogen, Carlsbad, CA), and then reverse-transcribed by M-MLV reverse transcriptase according to the manufacture's protocol (Invitrogen, CA). Real-time PCR was performed in the presence of Power SYBR green PCR Master Mix (TaKaRa, Japan) and the amplification was performed in ABI 7300 sequence detection system. *ZmGAPDH* and *AtActin* were used as internal control to normalize all data. The primers, used for evaluating the transcript levels of *ZmPMP3s, CSD1, CSD2, CSD3, CCS, APX1, APX2, P5CS1, P5CS2, CAT1, RD29B, DREB2A, KIN, PAB18, AVP1, AVP2, SOS1, HKT1, NHX1, ATK1, HKT1, CNGC12, AHA1, AHA2, AVA-P4*, in qRT-PCR experiment, were shown in Table S5. qRT-PCR analysis was performed at least three times using sets of cDNA samples from independent plants. Data from qRT-PCR experiments were analyzed according to the 2^−ΔΔCT^ method [66]. Specific primers were designed using PRIMER 5.0.

## Supporting Information

Figure S1 **Compilation of Kyte and Doolittle profiles for ZmPMP3s.** The ProtScale tool (http://web.expasy.org/protscale/) was used to analyze hydrophobic domains of the eight putative ZmPMP3 proteins.
(TIF)

Figure S2 **Subcellular localizations of remaining ZmPMP3-GFP fusion proteins in onion epidermal cell.** A) Fluorescent microscopic images of nonplasmolyzed cells transiently expressing ZmPMP3-GFP fusion proteins. B) Fluorescent microscopic images of plasmolyzed cells in 30% sucrose solution. A) Fluorescent microscopic images of GFP protein.
(TIF)

Table S1 **The other genes detected in** ***ZmPMP3-1*** **transgenic plants.**
(DOC)

Table S2 **Primers used for PCR amplifying full-length of the** ***ZmPMP3*** **genes.**
(DOC)

Table S3 **Primers used for subcellular localization of ZmPMP3s-GFP in onion epidermal cells.**
(DOC)

Table S4 **Primers used for cloning the** ***ZmPMP3*** **genes into pAUR123 vector.**
(DOC)

Table S5 **Primers used in qRT-PCR expression analysis of the** ***ZmPMP3*** **genes.**
(DOC)

Dataset S1 **Fasta file of all of the** ***ZmPMP3*** **CDS' nucleic acid sequences.** Accession numbers of sequences in GenBank were described in Table 1.
(FASTA)

Dataset S2 **Fasta file of putative proteins sequences of all** ***ZmPMP3*** **genes.** Calculation of the protein sequences was carried out by the tool of NCBI ORF finder (http://www.ncbi.nlm.nih.gov/gorf/gorf.html).
(FASTA)

Dataset S3 **Fasta file of genomic sequences of** ***ZmPMP3*** **genes in inbred line CN165.** The sequences were obtained from maize inbred line CN165, and these were used to analyze the gene structures of *ZmPMP3* genes.
(FASTA)
