# Supplementary figures and images for: Isolation and Characterization of Maize PMP3 Genes Involved in Salt Stress Tolerance
Source: PLoS One. 2012 Feb 13;7(2):e31101. doi: 10.1371/journal.pone.0031101 (PMC3278423; doi:10.1371/journal.pone.0031101)

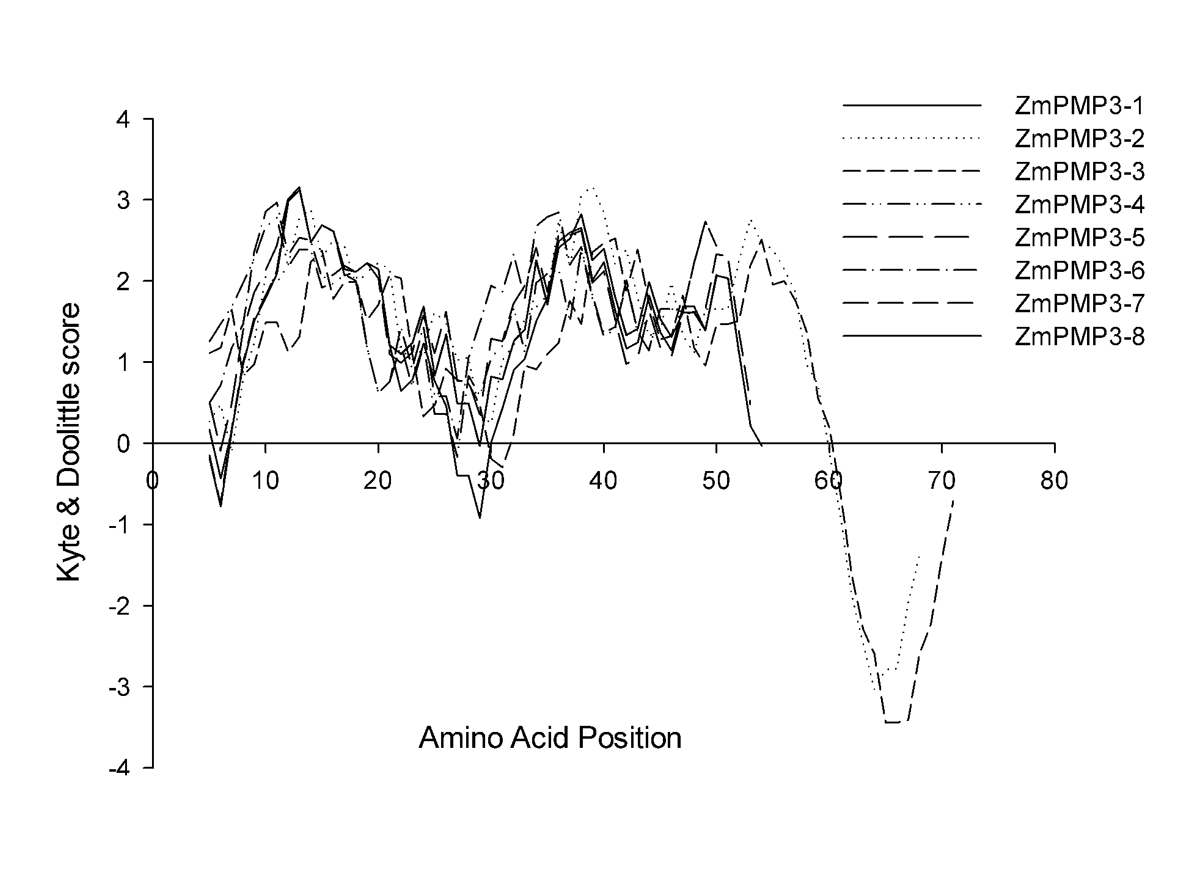

Supplement: Figure S1 — Compilation of Kyte and Doolittle profiles for ZmPMP3s. The ProtScale tool (http://web.expasy.org/protscale/) was used to analyze hydrophobic domains of the eight putative ZmPMP3 proteins. (TIF) [file pone.0031101.s001.tif]

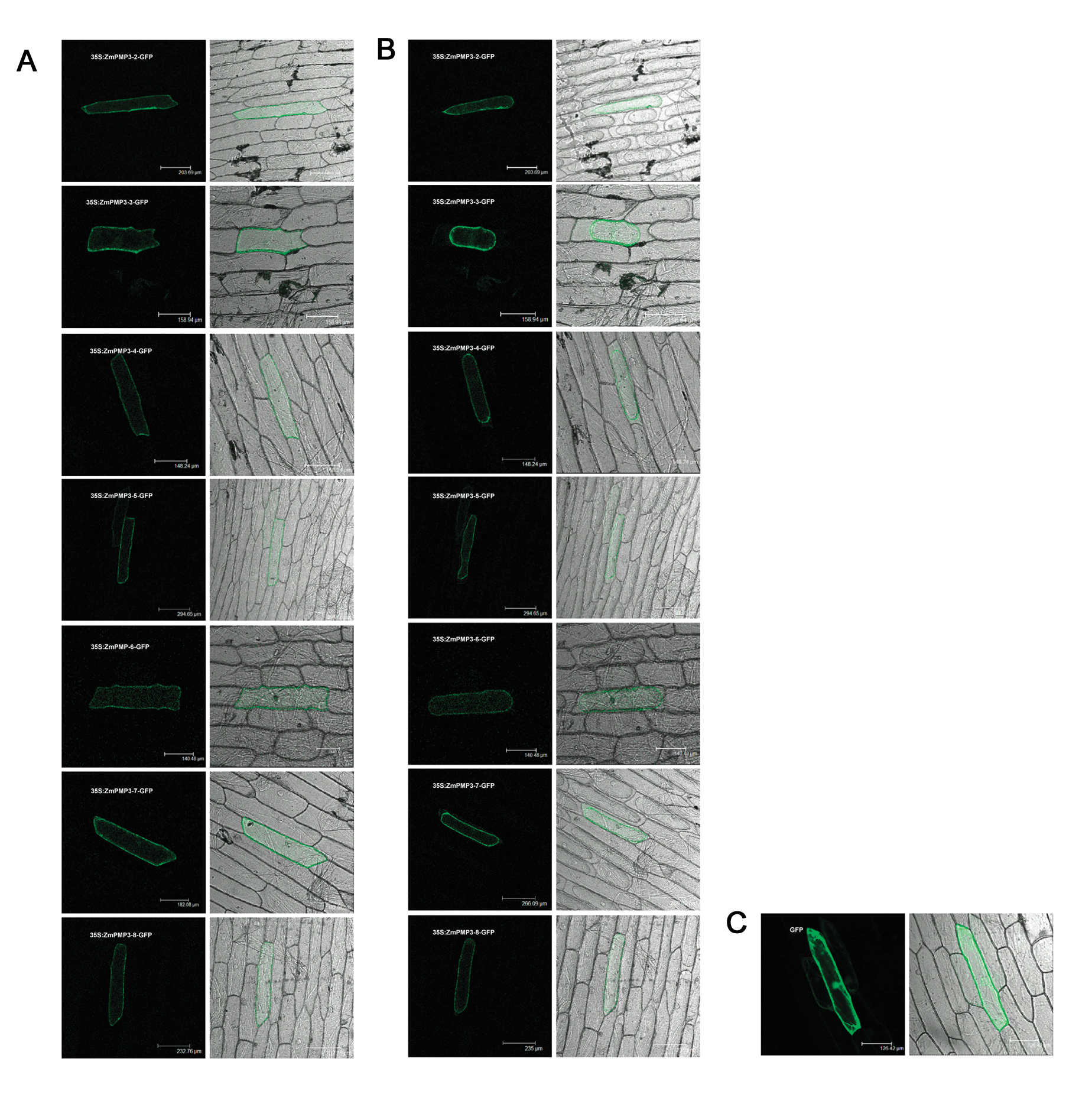

Supplement: Figure S2 — Subcellular localizations of remaining ZmPMP3-GFP fusion proteins in onion epidermal cell. A) Fluorescent microscopic images of nonplasmolyzed cells transiently expressing ZmPMP3-GFP fusion proteins. B) Fluorescent microscopic images of plasmolyzed cells in 30% sucrose solution. A) Fluorescent microscopic images of GFP protein. (TIF) [file pone.0031101.s002.tif]
