# Supplementary material for: Isolation and Characterization of Maize PMP3 Genes Involved in Salt Stress Tolerance
Source: PLoS One. 2012 Feb 13;7(2):e31101. doi: 10.1371/journal.pone.0031101 (PMC3278423; doi:10.1371/journal.pone.0031101)
Supplement: Table S1 — The other genes detected in ZmPMP3-1 transgenic plants. (DOC) [file pone.0031101.s003.doc]

**Table S1. The other genes detected in *ZmPMP3-1*** transgenic plants

| **Name*^a^*** | **Accession NO.*^b^*** | **Function annotation*^c^*** | **Relative expression1*^d^*** | **Relative expression2*^e^*** |
| --- | --- | --- | --- | --- |
| *CDS3* | NM_121815.3 | Superoxide dismutase3 | 0.877±0.077 | 1.091±0.028 |
| *APX1* | NM_001084012.1 | Ascorbate peroxidase 1 | 1.499±0.158 | 1.255±0.101 |
| *KIN* | NM_121601.2 | stress-induced protein KIN1 | 0.717±0.074 | 1.710±0.088 |
| *SOS1* | NM_126259.3 | Salt overly sensitive 1 | 1.051±0.091 | 0.825±0.025 |
| *HKT1* | NM_117099.5 | High-Affinity K+ transporter 1 | 0.530±0.042 | 0.911±0.05 |
| *AKT1* | NM_128222.5 | Arabidopsis K+ transporter 1 | 0.910±0.056 | 0.865±0.014 |
| *AVP2* | NM_001124147.1 | Arabidopsis vacuolar H+-Pyrophosphatase 2 | 1.470±0.089 | 0.781±0.052 |
| *CNGC12* | NM_180123.5 | Cation channel/cyclic nucleotide binding ion channel | 0.870±0.031 | 0.755±0.035 |
| *ATKT1* | NM_128562.3 | potassium ion transmembrane transporter | 0.573±0.035 | 0.831±0.018 |
| *AHA1* | NM_127453.3 | ATPase/ hydrogen-exporting ATPase | 1.608±0.271 | 0.753±0.109 |
| *AVA-P4* | NM_106215.3 | V-type proton ATPase proteolipid subunit c4 | 0.581±0.388 | 1.138±0.173 |

1. Name of the selected genes
2. Accession number of the selected genes in GenBank
3. Function annotation of the selected genes in GenBank.
4. Comparison of the relative expression levels of selected genes in *ZmPMP3-1* transgenic plants and WT under normal conditions.
5. Comparison of the relative expression levels of selected genes in *ZmPMP3-1* transgenic plants and WT under salt stress conditions.
