# Supplementary material for: Isolation and Characterization of Maize PMP3 Genes Involved in Salt Stress Tolerance
Source: PLoS One. 2012 Feb 13;7(2):e31101. doi: 10.1371/journal.pone.0031101 (PMC3278423; doi:10.1371/journal.pone.0031101)
Supplement: Table S2 — Primers used for PCR amplifying full-length of the ZmPMP3 genes. (DOC) [file pone.0031101.s004.doc]

**Table S2. Primers used for obtaining full-length of *ZmPMP3* genes**

| ZmPMP3-1 full F1 | AGCGAAAGGAGAGAAGGAATC |
| --- | --- |
| ZmPMP3-1 full R1 | GATGGGGTGGGTACGGTAG |
| ZmPMP3-2 full F1 | TCAGGAGCGAGGAAAGAGG |
| ZmPMP3-2 full R1 | GCCCAAGGTAGCACCAATC |
| ZmPMP3-3 full F1 | CGCCATCAATCAGCACTT |
| ZmPMP3-3 full R1 | CTCCACAGATCCCGCAAC |
| ZmPMP3-4 full F1 | GTTCCTCCTCCAGAGTCCAG |
| ZmPMP3-4 full R1 | ACAACATCCGCCGATAGTC |
| ZmPMP3-5 full F1 | AGGCTCTCTGATTTTACTTCTCC |
| ZmPMP3-5 full R1 | GACTTATTCCCATCGATCTCCCAAC |
| ZmPMP3-6 full F1 | TGCTTGCTACCACGCCT |
| ZmPMP3-6 full R1 | TAACTAAAGCAAGACACAAACATAC |
| ZmPMP3-7 full F1 | AAATTGTTGACAGTAGCGTGG |
| ZmPMP3-7 full R1 | TTCAGTCTCCATCCAGAAACAT |
| ZmPMP3-8 full F1 | TCAACCAGCGTGAACAAAC |
| ZmPMP3-8 full R1 | GCGGACCACTGAATAAATAC |
