# Supplementary material for: Isolation and Characterization of Maize PMP3 Genes Involved in Salt Stress Tolerance
Source: PLoS One. 2012 Feb 13;7(2):e31101. doi: 10.1371/journal.pone.0031101 (PMC3278423; doi:10.1371/journal.pone.0031101)
Supplement: Table S3 — Primers used for subcellular localization of ZmPMP3s-GFP in onion epidermal cells. (DOC) [file pone.0031101.s005.doc]

**Table S3**. Primers used for subcellular localization of ZmPMP3s-GFP in the onion epidermal cells.

| ZmPMP1-GFP-F1 | TATCTAGAATGTCGGAGGGGACTGCCAACTGCG |
| --- | --- |
| ZmPMP1-GFP-R1 | TAGCCGCGGTAGTTGTTCTTGGTGATGGCGTAG |
| ZmPMP2-GFP-F1 | CCAGCTCTagaATCGCGATGGCG |
| ZmPMP2-GFP-R1 | CCACCGCGGCAAGCAAGGGTG |
| ZmPMP3-GFP-F1 | CAGGTctAGaAAGGAGATGGGCCTG |
| ZmPMP3-GFP-R1 | TCCCCGCGGCATGCGACGTAG |
| ZmPMP4-GFP-F1 | GAGAtctAGAGGCAATGGCGTCGG |
| ZmPMP4-GFP-R1 | TCGCCCGCGGTAAGCCACCAGC |
| ZmPMP5-GFP-F1 | CACGtcTAGAAAAGCAAGATGTCGGA |
| ZmPMP5-GFP-R1 | TCCGCCGCGGTACTTGGTGATG |
| ZmPMP6-GFP-F1 | GGCGTCtAgAATGGGGTCGGAGAC |
| ZmPMP6-GFP-R1 | TTGCCGCGGCACGCGACGAG |
| ZmPMP7-GFP-F1 | ATCGTCTAGAGGGGAGGGAGAGAGAT |
| ZmPMP7-GFP-R1 | GCTACCGCGGTAGTCCTTGGTGATA |
| ZmPMP8-GFP-F1 | GAGATCTAGAAAGATGAAGGAGGGCACG |
| ZmPMP8-GFP-R1 | CGCCCGCGGTAGTCCTTGGTG |
