# Supplementary material for: Isolation and Characterization of Maize PMP3 Genes Involved in Salt Stress Tolerance
Source: PLoS One. 2012 Feb 13;7(2):e31101. doi: 10.1371/journal.pone.0031101 (PMC3278423; doi:10.1371/journal.pone.0031101)
Supplement: Table S4 — Primers used for cloning the ZmPMP3 genes into pAUR123 vector. (DOC) [file pone.0031101.s006.doc]

**Table S4. Primers used for cloning *ZmPMP3* genes into pAUR123 vector.**

| ZmPMP1-PAUR-F1 | TGGGGTACCAAGAAGCATCGGCAGCAT |
| --- | --- |
| ZmPMP1-PAUR-R1 | TATGAGCTCACGCAGATACAGAGACAT |
| ZmPMP2-PAUR-F1 | TCCGGTACCATCCGTCCCGATCCCG |
| ZmPMP2-PAUR-R1 | GGCGAGCTCAGATGCTCAAGCAAGGGTGT |
| ZmPMP3-PAUR-F1 | GTCGGTACCAGGTGGAGGAAGGAGATG |
| ZmPMP3-PAUR-R1 | GACGAGCTCCAGAGCACCACGGGGAC |
| ZmPMP4-PAUR-F1 | GAGGGTACCAGAGAGGCAATGGCGTCG |
| ZmPMP4-PAUR-R1 | CTTGAGCTCCTCCTCTTCGCACAACTTAA |
| ZmPMP5-PAUR-F1 | GAAGGTACCGATGTCGGACGGCACGG |
| ZmPMP5-PAUR-R1 | GCAGAGTCCGGAGTCATTGCAGGGGG |
| ZmPMP6-PAUR-F1 | CTAGGTACCGGCGGCTTTGGCG |
| ZmPMP6-PAUR-R1 | CGGGAGTCCACCATCGTCGTCATACCTAC |
| ZmPMP7-PAUR-F1 | CAAGGTACCAGAGGGGAGGGAGAGAGAT |
| ZmPMP7-PAUR-R1 | GTTGAGCTCGCTATCTGGCTAGTCCTTGGT |
| ZmPMP8-PAUR-F1: | AGAGGTACCAAGATGAAGGAGGGCAC |
| ZmPMP8-PAUR-R1 | GCAGAGCTCACCCAACTGGAACCG |
