# Supplementary material for: Isolation and Characterization of Maize PMP3 Genes Involved in Salt Stress Tolerance
Source: PLoS One. 2012 Feb 13;7(2):e31101. doi: 10.1371/journal.pone.0031101 (PMC3278423; doi:10.1371/journal.pone.0031101)
Supplement: Table S5 — Primers used in qRT-PCR expression analysis of the ZmPMP3 genes. (DOC) [file pone.0031101.s007.doc]

**Table S5. Primers used for qRT-PCR expression analysis of *ZmPMP3* genes and selected stress-related genes.**

| ZmPMP1-RT-S1 | CAACTGCGTGGACATCCTGA |
| --- | --- |
| ZmPMP1-RT-A1 | GGCGTAGATGGCAGTGATGA |
| ZmPMP2-RT-S1 | AGGAGCGAGGAAAGAGGGAG |
| ZmPMP2-RT-A1 | GGGAGGATGATGGCGAAG |
| ZmPMP3-RT-S1 | GCCGCAGGTGGAGGAAGG |
| ZmPMP3-RT-A1 | GGTAGCCGAGGATGGTGAGCA |
| ZmPMP4-RT-S1 | AGTTCTGGATCGACCTCTTGCTG |
| ZmPMP4-RT-A1 | ACATCCGCCGATAGTCACTCCT |
| ZmPMP5-RT-S1 | TTCTGGATCTGCCTTATCCTCAC |
| ZmPMP5-RT-A1 | GATTTCTTCAGCCGAGCAAACT |
| ZmPMP6-RT-S1 | TGCTTGCTACCACGCCTCCT |
| ZmPMP6-RT-A1 | ACGAACGTCTCCGACCCCAT |
| ZmPMP7-RT-S1 | ATCGTCATCAATCGAGCAGAGG |
| ZmPMP7-RT-A1 | CGCTATCTGGCTAGTCCTTGGTG |
| ZmPMP8-RT-S1 | GCAAGATGAAGGAGGGCACG |
| ZmPMP8-RT-A1 | TCTCTCACCCAACTGGAACCG |
| P5CS1-F(898) | GTTGCTGCGAGGGAAAGT |
| P5CS1-R(1076) | AAGCGAGCCACCATTGAC |
| P5CS2-F(1598) | CCGATGCAAAGTTGGACTATCC |
| P5CS2-R(1801) | CCTTGGAACTGTACTCGTGGTG |
| APX1-F(38) | AGAAGGCTGTTGAGAAGTGC |
| APX1-R(240) | CAACCTAAGAGCAATGTGGAT |
| CCS-F(108) | CAACCCCAAATCTCAATCTCTC |
| CCS-R(361) | AATCCACCTCCACTTTCTCAATAC |
| CDS1-F(35) | AGGGTGTTACGGGGACTATCT |
| CDS1-R(203) | TTACCATCGGGGTTGAAATG |
| CSD2-F(49) | CTCATTCCTCCTTCCTCCA |
| CSD2-R(181) | CAACTGTCAACGCTTTCGA |
| CSD3-F(251) | GTCATGCTGGCGATTTGG |
| CSD3-R(412) | GCTTGTGCCCTCCTTTCC |
| CAT1-F(728) | TTGGAGGAGCCAATCACAGC |
| CAT1-R(924) | CAAGACCAAGCGACCAACAG |
| SOS1-F(1635) | GATGCTTGATGAGGGCAGAAT |
| SOS1-R(1827) | GACAGCAAAGTATGTGACCAACT |
| NHX1-F(424) | GACTTGGGTGATTATCTTGCTATTG |
| NHX1-R(622) | CAGCTTCGTGGTTTAGGTGAG |
| HKT1-F(783) | GCGTGACGAGTATGGTTACATTCT |
| HKT1-R(1000) | GCGAATTCACCACTTGAAACAAC |

**Table S5 (continued)**

| RD29B-F(373) | ACGACGGAAACATCGGACT |
| --- | --- |
| RD29B-R(552) | TTCACCACCAGGAGCAAAC |
| RAB18-F(269) | GCCAAGAGCAACTCCACAAG |
| RAB18-R(426) | ATGATGACCTGGCAACTTCT |
| KIN-F(66) | CAATGTTCTGCTGGACAAGG |
| KIN-R(152) | GCCGCATCCGATACACTCT |
| DREB2A-F(195) | TATGAAAGGTAAAGGAGGACCAG |
| DREB2A-R(343) | CAGCTTCTTGAGCAGTAGGG |
| HKT1-F(783) | GCGTGACGAGTATGGTTACATTCT |
| HKT1-R(1000) | GCGAATTCACCACTTGAAACAAC |
| CNGC12-F(24) | TGCCAGGATAGATAGTATGGGAGT |
| CNGC12-R(191) | ATCAAAGGGATAAACAGAAACAAA |
| AVP1-F(1709) | ACACCGTAGATGTTTTGACCCC |
| AVP1-R(1881) | TGCGGTTCCTTCCATAAGTCC |
| AVP2-F(1977) | GGAGTACAAGGAGAAGCCAGATT |
| AVP2-R(2120) | GTGTAGTAACCCAAGATTCGAAAC |
| AVA-P4-F(240) | GATTAACCCCAAGGCCAAGT |
| AVA-P4-R(359) | GCATCACCGACGATTCCA |
| ATKT1-F(669) | TTTCGCTCCAATCTCCACTG |
| ATKT1-R(826) | CTCCAAGTGAAACCCACCCT |
| AKT1-F(554) | ACTACTTTTGGGTCCGATGC |
| AKT1-R(669) | CCAGGTCTTTGCGGGGTT |
| AHA2-F(1691) | TCTTCCCAGAGCACAAATACGA |
| AHA2-R(1891) | CACTGATAATAACGCTGAGTCCAG |
| AHA1-F(1361) | GTCTTAGGTCGTTGGCAGTAGC |
| AHA1-R(1478) | CTGTCGTGTCTTGGAGGGTC |
